# Long oligodeoxynucleotides: chemical synthesis, isolation via catching-by-polymerization, verification via sequencing, and gene expression demonstration

**DOI:** 10.3762/bjoc.19.146

**Published:** 2023-12-21

**Authors:** Yipeng Yin, Reed Arneson, Alexander Apostle, Adikari M D N Eriyagama, Komal Chillar, Emma Burke, Martina Jahfetson, Yinan Yuan, Shiyue Fang

**Affiliations:** 1 Department of Chemistry and Health Research Institute, Michigan Technological University, 1400 Townsend Drive, Houghton, MI 49931, USA; 2 College of Forest Resources and Environmental Science, Michigan Technological University, 1400 Townsend Drive, Houghton, MI 49931, USA

**Keywords:** automated synthesis, catching-by-polymerization, gene assembly, long oligonucleotide, synthetic biology

## Abstract

Long oligodeoxynucleotides (ODNs) are segments of DNAs having over one hundred nucleotides (nt). They are typically assembled using enzymatic methods such as PCR and ligation from shorter 20 to 60 nt ODNs produced by automated de novo chemical synthesis. While these methods have made many projects in areas such as synthetic biology and protein engineering possible, they have various drawbacks. For example, they cannot produce genes and genomes with long repeats and have difficulty to produce sequences containing stable secondary structures. Here, we report a direct de novo chemical synthesis of 400 nt ODNs, and their isolation from the complex reaction mixture using the catching-by-polymerization (CBP) method. To determine the authenticity of the ODNs, 399 and 401 nt ODNs were synthesized and purified with CBP. The two were joined together using Gibson assembly to give the 800 nt green fluorescent protein (GFP) gene construct. The sequence of the construct was verified via Sanger sequencing. To demonstrate the potential use of the long ODN synthesis method, the GFP gene was expressed in *E. coli*. The long ODN synthesis and isolation method presented here provides a pathway to the production of genes and genomes containing long repeats or stable secondary structures that cannot be produced or are highly challenging to produce using existing technologies.

## Introduction

Long oligodeoxynucleotides (ODNs) are segments of DNAs extending beyond one hundred nucleotides (nt). Emerging research areas such as synthetic biology [1–2], protein engineering [3–4], mRNA vaccine [5], CRISPR/Cas9 genome editing [6], and DNA digital information storage [7] are constrained by the lack of affordable and high-quality long ODNs with no or little sequence restrictions and short turnaround time. Currently, long ODNs are mostly assembled using chemically synthesized ODNs of 20–60 nt in length using PCR or ligation [8]. While these methods have made possible the emergence of research areas such as synthetic biology [9–14], they have various drawbacks [3]. (1) Owing to the short length of the starting ODNs, a large number of them are needed in the PCR assembly step. Because of this, misalignments may occur, and errors may be introduced. (2) PCR assembly of multiple short ODNs in one pot requires similar melting points of the multiple overlapping regions of the short ODNs. Therefore, the sequences of the short ODNs must be carefully chosen via means such as codon swapping. However, this is not always easy to do, and an error may fail the entire assembly. (3) Stable secondary structures are usually troublesome for PCR. For this reason, the GC content of the target long ODN has to be restricted to a limited range because high GC content is usually associated with promiscuous annealing and secondary structures. Problematically, many secondary structures such as hairpin, cruciform, and G-quadruplex are actually needed for genes and genomes to function, and thus these genes and genomes may not be accessible. (4) Genes having long repeats widely appear in many genomes [15]. These sequences cannot be assembled using ODNs shorter than the repeats. (5) Long turnaround time, intense labor, and high costs. The long turnaround time is particularly problematic for synthetic biology because to answer a biological question or to produce a desired functional organism, multiple cycles of design-build-test-learn are required, and in each cycle a new genome is needed [16]. (6) Inability to access long ODNs that contain site-specific modifications such as 5-methyldeoxycytidine (5mC) and 6-methyldeoxyadenosine (6mA). A method for direct automated de novo synthesis of long ODNs would solve or alleviate many of these problems.

Significant efforts have been made toward de novo synthesis of long ODNs through engineering of template-independent terminal deoxynucleotidyl transferase (TdT) [17]. While promising, this enzymatic method is unlikely to address all the aforementioned problems, such as generating sequences having highly stable secondary structures [18]. In this paper, we report a method to access long ODNs involving automated direct de novo chemical synthesis and isolation of full-length sequences using the catching-by-polymerization (CBP) technique [19]. Using the method, long ODNs with 399 and 401 nt were directly synthesized on an automated synthesizer. The full-length sequences were extracted from the complex mixture generated from over a thousand reactions using CBP. The two ODNs were joined together to form the 800 nt green fluorescent protein (GFP) gene construct using Gibson assembly. Sanger sequencing confirmed the authenticity of the long ODNs. To demonstrate the applications of the long ODN synthesis method, the GFP gene was expressed in *E. coli*.

## Results

### Long ODN synthesis and CBP purification

**Figure 1:**
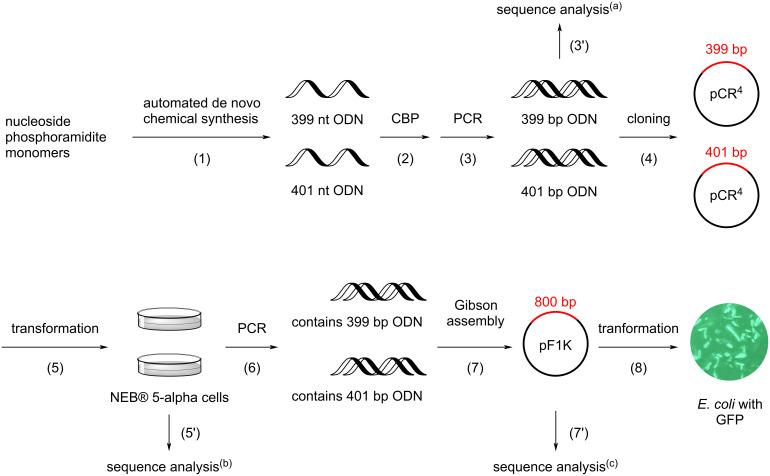
Workflow for the construction, verification, and expression of the 800 bp GFP gene from chemically synthesized long ODNs. (a) Sequence analysis via gel electrophoresis by comparing with control ODNs (see Figure 2A,B). (b) Sequence analysis via colony PCR, gel electrophoresis (see Figure 3), and Sanger sequencing. (c) Sequence analysis via transforming NEB^®^ 5-alpha cells, colony PCR, gel electrophoresis (Figure 2C), and Sanger sequencing. CBP, catching-by-polymerization. GFP, green fluorescent protein. nt, nucleotide. PCR, polymerase chain reaction.

**Scheme 1:**
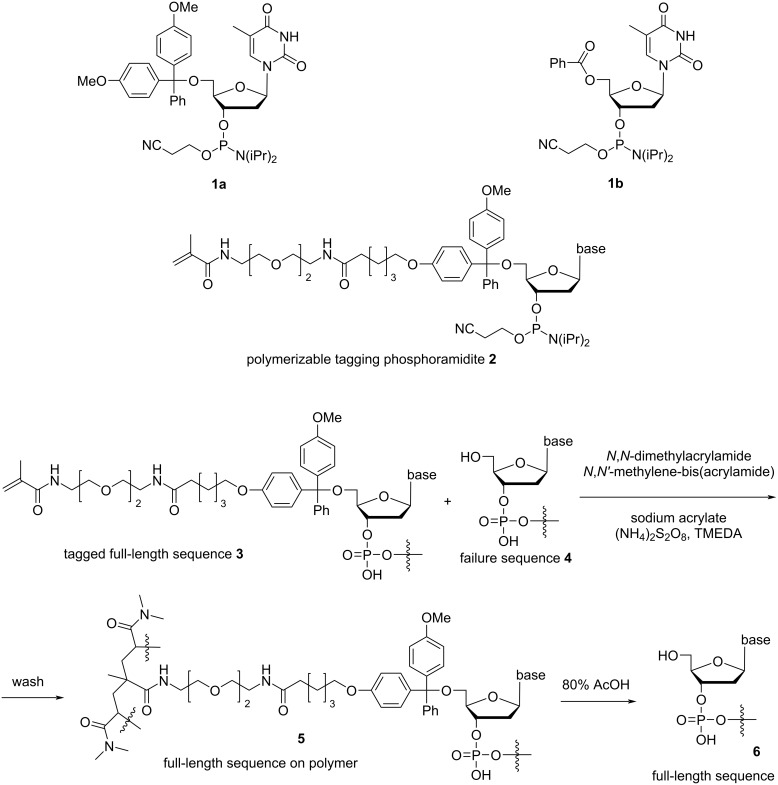
Structure of phosphoramidites **1a**,**b** and **2**, and the catching-by-polymerization (CBP) process.

The 800 nt (all ODN sequences are provided in Supporting Information File 1) GFP gene construct was divided into a 399 and 401 nt ODNs for automated synthesis (step 1, Figure 1). The syntheses were carried out in commercial 0.2 µmol 2000 Å CPG columns on an ABI 394 DNA/RNA synthesizer using phosphoramidite chemistry. To ensure full coverage of CPG in the column by reagent solutions in each of the over one thousand reactions, the synthesizer manufacturer’s recommendation of 1 µmol instead of 0.2 µmol for standard synthetic cycle was used with several minor but critical modifications (see Supporting Information File 1 for details). To block any hydroxy groups on the surface of CPG that were not capped, including those resulted from mechanical breakage of CPG, before commencing a synthesis, the CPG was subjected to typical capping conditions for 20 minutes. To reduce steric hindrance within the pores of CPG caused by long ODNs, the loading of the synthesis column was reduced from 0.2 µmol to 0.1 µmol. This was achieved by applying a solution containing 0.05 M 5'-DMTr-dT-phosphoramidite (**1a**, Scheme 1) and 0.05 M 5'-Bz-dT-phosphoramidite (**1b**) for the coupling step in the first synthetic cycle. Because the Bz group is stable during deblocking, approximately half of the active sites of the CPG were blocked. Parameters for the four steps of the synthetic cycle were as recommended by the manufacturer, except for the coupling step where the waiting time was increased from 25 to 35 seconds. In the last synthetic cycle, the polymerizable tagging phosphoramidite **2**, of which the synthesis was described earlier [20] (Scheme 1), was used in the coupling step, and the reaction was repeated three times for 5 minutes each time to maximize the yield of tagging the full-length sequences with the polymerizable methacrylamide group. Detritylation was not carried out in the last synthetic cycle, which would otherwise remove the polymerizable tag.

A portion of the CPG was subsequently subjected to deprotection and cleavage. To prevent the potential Michael addition side reaction of acrylonitrile to nucleobases, the 2-cyanoethyl groups were removed by flushing the CPG with a solution of DBU in ACN. Under these conditions, the ODN remains on CPG and the nucleobases remain protected, both of which decrease the probability of the Michael addition side reaction. After washing off acrylonitrile, the CPG was subjected to typical deprotection and cleavage conditions using concentrated ammonium hydroxide at elevated temperature. At this stage, the mixture contained the desired full-length sequence **3** and impurities such as failure sequences **4** and small molecules from ODN deprotection (Scheme 1). It is noted that the full-length sequence **3** contains a polymerizable methacrylamide group at its 5'-end; the failure sequences **4** and many other impurities do not.

In principle, due to the large relatively hydrophobic tag introduced by **2**, the full-length sequence could be isolated by RP HPLC. However, we found that this is impossible [20]. Once the length of ODNs reaches about 100-mer, RP HPLC could not resolve the full-length and failure sequences. Therefore, to isolate the full-length sequence, the catching-by-polymerization (CBP) method was employed (step 2, Figure 1, and Scheme 1). To the crude ODN, the polymerization monomers *N*,*N*-dimethylacrylamide and sodium acrylate, and the cross-linking agent *N*,*N'*-methylenebisacrylamide were added, and the polymerization was initiated with ammonium persulfate and *N*,*N*,*N'*,*N'*-tetramethylethylenediamine (TMEDA). The full-length sequence **3** was co-polymerized into the polymer **5**, and failure sequences **4** and other impurities were then washed away using aqueous solutions including mildly basic solutions. These washes might also remove *N*,*N*-dimethylacrylamide added to ODNs via Michael addition. The full-length ODN **6** was cleaved from the polyacrylamide gel (**5**) using 80% acetic acid (Scheme 1). Because depurination is less likely for deprotected ODNs, and the conditions are widely used for detritylation after RP HPLC, ODN damage in this step is unlikely or negligible. The purified ODNs, 399 and 401 nt, were each extracted from their gel. To remove residual acetic acid, the ODNs were dissolved in water and precipitated with *n-*BuOH. This gave the CBP-purified ODNs.

### Analysis of long ODNs with gel electrophoresis and Sanger sequencing

**Figure 2:**
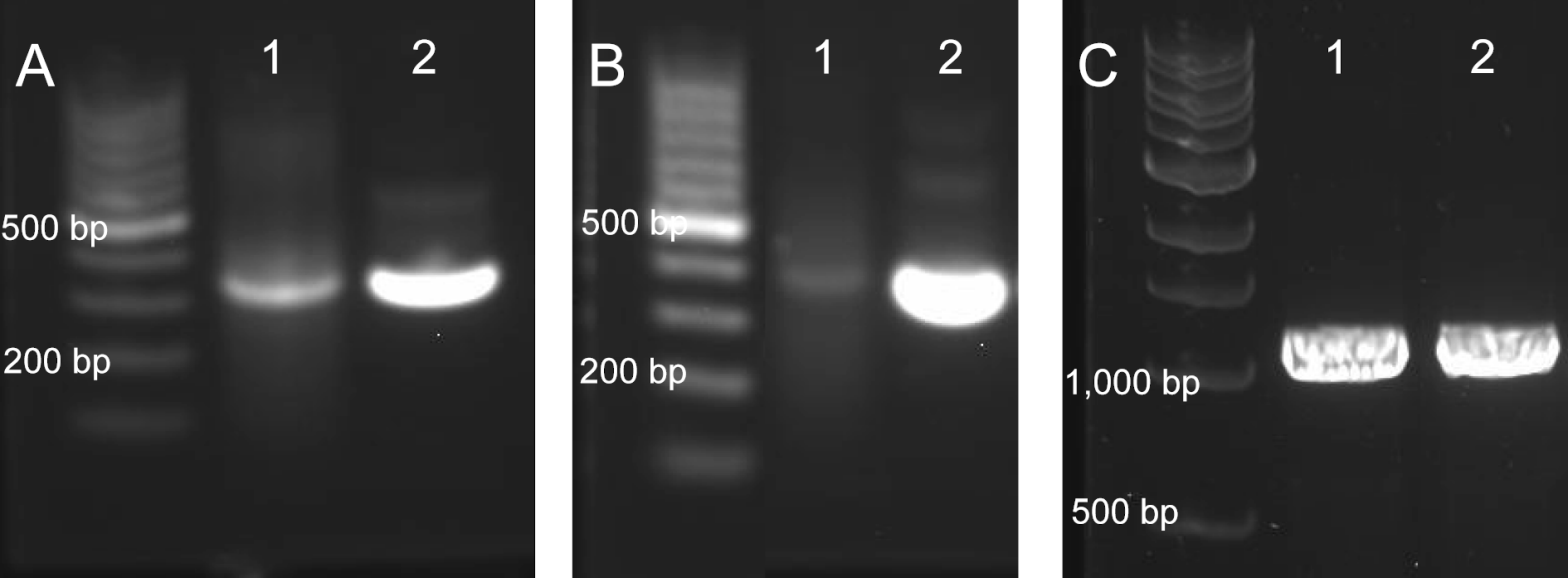
Gel electrophoresis images of 399 bp (A), 401 bp (B), and 800 bp (C) dsODNs derived from synthetic 399 and 401 nt ssODNs. (A) Lane 1: 399 bp dsODN obtained with PCR using 399 nt ssODN as template. The ssODN was synthesized de novo on an automated synthesizer and purified with CBP. Lane 2: control 399 bp dsODN obtained with PCR using commercial 800 bp dsODN GFP gene and appropriate primers. Agarose gel (1%) electrophoresis and GelRed^®^ staining were used to obtain the image. (B) Lane 1: 401 bp dsODN obtained as described above for 399 bp dsODN. Lane 2: control 401 bp dsODN obtained as described above for 399 bp dsODN control. Gel image was obtained under the same conditions for (A). (C) Lane 1: dsODN (800 bp plus primers) obtained from synthetic 399 nt and 400 nt ssODNs as described in Figure 1 and experimental section. Lane 2: control dsODN (800 bp plus primers) obtained from commercial 800 bp dsODN GFP gene as described in the experimental section. Agarose gel (2%) electrophoresis and GelRed^®^ staining were used to obtain the image.

One simple method to estimate the length of the long ODNs would be to compare them with authentic samples using gel electrophoresis. However, commercial suppliers were not able to provide single-stranded ODNs (ssODNs) with this length. Because double-stranded ODNs (dsODNs) of a few hundred nucleotides in length devoid of difficult structures are relatively easy to access using well-established technologies such as PCR assembly, and they are available from commercial sources, we converted our synthetic 399 and 401 nt ssODNs to dsODNs with PCR. For authentic dsODNs, we purchased the 800 bp GFP gene from a commercial source. Using the gene as the template, the authentic 399 and 401 bp dsODNs were obtained with PCR. The dsODNs derived from synthesized ssODNs were compared with the authentic dsODNs using agarose gel electrophoresis (steps 3 and 3', Figure 1). As shown in Figure 2A,B, the synthesized and authentic samples have similar migration distances.

**Figure 3:**
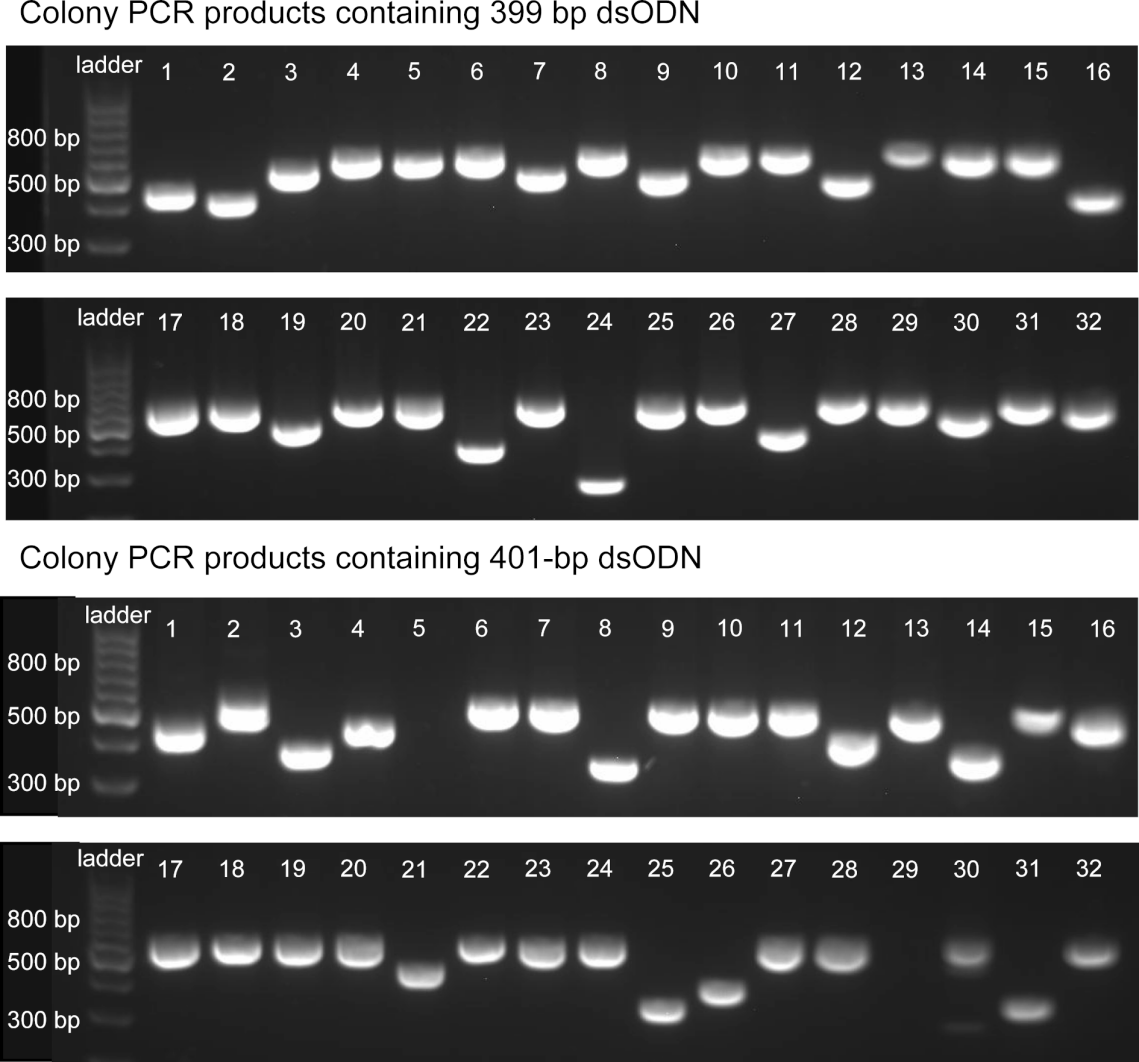
Gel electrophoresis images of ≈399 and ≈401 bp dsODNs originated from synthetic 399 and 401 nt ssODNs. The chemically synthesized and CBP-purified 399 and 401 nt ssODNs were converted to dsODNs by PCR. The dsODNs were then cloned into pCR4 vector and transformed into NEB^®^ 5-alpha cells. In each of the 399 and 401 nt ssODN cases, 32 colonies were selected for colony PCR using M13 primers, which covered the synthetic 399 and 401 bp ODN regions. The colony PCR products were analyzed with 2% agarose gel electrophoresis. The gels were stained with GelGreen^®^. The M13 primers covered an additional 240 bp, and therefore the PCR products containing the full-length 399 bp and 401 bp dsODNs have about 640 bp, and those without incorporated dsODNs have about 240 bp. For the case of 399 bp dsODN, colonies corresponding to lanes 4–6, 8, 10, 11, 13–15, 17, 18, 20, 21, 23, 25, 26, 28, 29, 31, and 32 probably had the 399 bp dsODN. Colonies of lanes 13, 15, and 26 were submitted for Sanger sequencing; 13 and 15 had errors, and 26 had the correct 399 bp sequence. For the case of 401 bp dsODN, colonies corresponding to lanes 2, 6, 7, 9–11, 13, 15, 17–20, 22–24, 27–28, 30, and 31 probably had the 401 bp dsODN. Colonies of lanes 6, 10, and 20 were submitted for Sanger sequencing; 6 and 20 had errors, and 10 had the correct 401 bp sequence. Detailed sequence alignments are provided in Supporting Information File 1.

The identity of the ODNs was then determined by Sanger sequencing. The PCR products were cloned into the pCR™4Blunt-TOPO™ vector (step 4, Figure 1), and transformed into NEB^®^ 5-alpha chemically competent cells (step 5). The transformed cells were plated on agar plates containing kanamycin and grown overnight. For each construct, the insert of 32 colonies was amplified with colony PCR using vector M13 forward and reverse primers (step 6). The PCR products were analyzed with agarose gel electrophoresis (step 5', Figure 1, and Figure 3). Based on migration distances, in the case of 399 bp sequence, 20 out of 32 colonies likely contained the expected 399 bp fragment. Three colonies were submitted for Sanger sequencing (step 5', Figure 1). One colony (the one corresponding to lane 26) was found to have the correct full-length 399 bp sequence (see Supporting Information File 1 for sequence alignments). The sequences in the other two colonies had errors with one containing a dG-to-dA substitution, and the other containing one single-nucleotide (dA) deletion. In the case of the 401 bp sequence, 19 out of 32 colonies contained the expected 401 bp fragment (Figure 3). Three colonies were submitted for Sanger sequencing (step 5', Figure 1). One colony (the one corresponding to lane 10) was found to have the correct full-length sequence. The sequences in the other two colonies had errors with one containing one dG-to-dA substitution and the other containing one single-nucleotide (dC) deletion, and one dT-to-dC and one dG-to-dA substitution.

### Construction of GFP gene and GFP expression in *E. coli*

Gibson assembly was employed to assemble the 399 and 401 bp dsODNs, which were originated from the chemically synthesized 399 and 401 nt ssODNs, into the pF1k T7 Flexi^®^ vector. To do this, the pF1k vector was cut with PmeI and SgfI restriction enzymes. The linearized vector was purified with agarose gel electrophoresis. The 399 and 401 bp fragments from sequencing confirmed clones were elongated with PCR using appropriate primers (see Supporting Information File 1 for details) so that the fragments have homologous overlapping ends with each other and with the insertion sites of the vector. The PCR elongated ≈399 and ≈401 bp fragments were also purified with agarose gel electrophoresis. The linearized pF1k vector and the elongated ≈399 and ≈401 bp dsODNs were subjected to typical Gibson Assembly conditions (step 7, Figure 1, see Supporting Information File 1 for details) [21]. The recombinant vector containing the full-length 800 bp GFP gene construct was transformed into NEB^®^ 5-alpha chemically competent cells (step 7', Figure 1). The transformed cells were plated on kanamycin plates and grown at 37 °C overnight. Colony PCR was carried out over selected colonies, and the PCR products were analyzed with agarose gel electrophoresis. A band was observed above the 800 bp region indicating that the assembly likely succeeded (lane 1, Figure 2C). Sanger sequencing of two colonies further confirmed that the colonies contained the correct sequence of the 800 bp dsODN (see Supporting Information File 1 for sequence alignments). Plasmid DNAs from these confirmed clones were used for GFP expression experiments described below. The authentic 800 bp dsODN GFP gene from commercial source was also assembled into the pF1k vector using Gibson assembly and transformed into NEB^®^ 5-alpha cells. Colony PCR and agarose gel electrophoresis indicated that the assembly succeeded (lane 2, Figure 2C). Plasmid DNAs of two positive colonies were submitted for Sanger sequencing. One clone was confirmed to contain the correct GFP gene construct, and the construct was in frame with the expression vector. The other clone was found to contain a dG-to-dA substitution error (see Supporting Information File 1 for sequence alignments).

**Figure 4:**
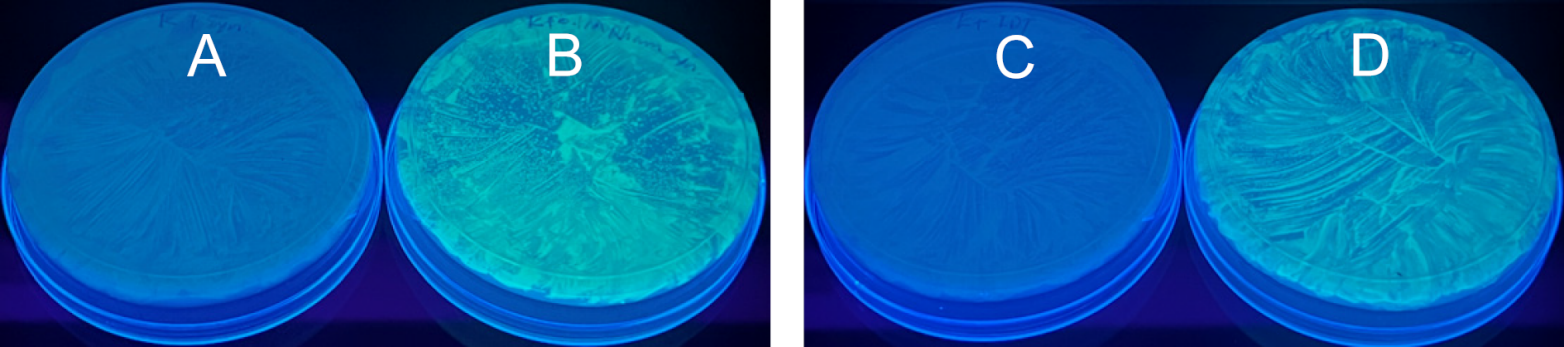
Images of *E. coli* containing the GFP gene. (A) The GFP gene was derived from the 399 and 401 nt chemically synthesized ODNs and isolated using the CBP method. Rhamnose was not added, and GFP was not expressed. (B) The same as (A) except that rhamnose was added, and GFP was expressed. (C) The GFP gene was from a commercial source. Rhamnose was not added, and GFP was not expressed. (D) The same as (C) except that rhamnose was added, and GFP was expressed. The results indicate that the gene derived from synthetic long ODNs functions in an identical manner as the control gene from the commercial source.

Plasmid DNAs, which were isolated from the clones with sequencing confirmed full length GFP gene, were transformed into *E. coli* Single Step (KRX) competent cells, which allow for tightly controlled gene expression by requiring rhamnose induction, were used for the transformation (step 8, Figure 1). The cells were plated on an agar plate containing kanamycin and incubated at 37 °C overnight. Cells from a single colony on the plate were incubated in LB containing kanamycin. A portion of the culture was plated on an agar plate containing kanamycin and rhamnose. As shown in Figure 4, GFP was successfully expressed. As a negative control, a portion of the culture was also plated on an agar plate containing kanamycin but not rhamnose. As expected, fluorescence of GFP was not observed. The same gene expression procedure was also applied to plasmid DNAs with sequencing confirmed GFP gene from commercial source. As shown in Figure 4, GFP expression occurred on the plate with rhamnose, and did not occur on the plate without rhamnose. The results confirmed that the 800 bp gene was successfully derived from the 399 and 401 nt long synthetic ssODNs, and functional in biological systems.

## Discussion

For ODN purification using CBP, one major concern has been the damage of the ODN under the radical acrylamide polymerization conditions. Although some work has been done to prove that this is unlikely [22–25], direct evidence is lacking in the literature. For the CBP method to be practically useful, we believe that characterization of the ODNs via DNA sequencing is imperative. Based on this reasoning, ODNs from three colonies originated from the 399 nt GFP gene fragment were sequenced (see Supporting Information File 1 for sequence alignments). One of them contained the correct full-length 399 bp ODN. The other two had errors, among which one contained a dG-to-dA substitution, and the other contained a single-nucleotide (dA) deletion. For the 401 nt GFP fragment, ODNs from three colonies were sequenced as well. One contained the correct sequence, one contained a dG-to-dA substitution, and the third one contained a single-nucleotide (dC) deletion, and a dT-to-dC and dG-to-dA substitution. The dG-to-dA substitution is one of the most common errors in synthetic ODNs [26]. Its occurrence is likely due to the formation of 2,6-diaminopurine from a capping-modified guanine base during ammonium hydroxide ODN deprotection and subsequent reading as dA during PCR [26–28]. The dT-to-dC substitution could have been resulted from amination of dT to give 5-methyl-dC, which is recognized as dC in PCR as well as sequencing [26]. Single-nucleotide deletion could be a result of failure sequences that were not capped in the capping step or incomplete detritylation. Plasmids of two colonies containing the GFP gene assembled from the 399 and 401 bp fragments were also submitted for Sanger sequencing. No error was found. As a control, ODNs of two colonies containing the GFP gene construct cultivated using commercial GFP gene were also submitted for Sanger sequencing. One sequence was correct. One had a dG-to-dA substitution error (see Supporting Information File 1 for sequence alignments). The sequencing results further confirmed that ODN damage by radicals during CBP purification is unlikely.

Another concern for long ODN synthesis is the perceived low quantity of product [29]. For example, for a 400 nt ODN synthesis, if the average stepwise yield is 99.0% as usually observed in trityl assay for the synthesis of ODNs with around 30 nt, the total yield of full-length ODN would only be about 1.8%. For a 0.1 µmol synthesis, the quantity of product would be 1.8 nmol. This is not too bad as long as it can be isolated because the quantity is more than the 1 pmol quantity mentioned by other researchers for some biological applications [30–31]. Besides quantity, for long ODN synthesis, the accuracy of the product is also important. According to reported data [26], common errors in synthetic ODNs include substitution, insertion, and deletion, and their rates are 0.045% (dG to dA 0.11%, dT to dC 0.01%, dC to dT 0.02%, dA to dG 0.01%, dG to dT 0.03%; 0.18/4; equal occurrence rate of the four nucleotides in ODNs is assumed), 0.0045% (dG 0.008%, dA 0.005%, dC 0.003%, dT 0.002%; 0.018/4), and 0.1%, respectively. Based on these data, the chance for the identity of a nucleotide in a specific position in a sequence to be correct is 99.8505% (100% − 0.1495%). Therefore, for a 400 nt ODN synthesis a product molecule has a chance of 55% (0.998505^400^) to have the correct sequence. This is still not too bad because statistically among two colonies containing the long ODN product, one would be likely to have the correct sequence.

In the present case, we did the 399 and 400 nt ODN syntheses at the scale of 0.1 µmol. One tenth of the CPG was subjected to deprotection and cleavage (theoretically 10 nmol). One fourth was subjected to CBP (theoretically 2.5 nmol). Quantification of 1/160 (theoretically 15.625 pmol) indicated that it contained about 40 ng (0.14 pmol) ODN, which corresponds to a total yield of about 1% for the entire process of ODN synthesis, deprotection and cleavage, CBP purification, and other steps. The result is consistent with a stepwise yield of about 98.9% for ODN synthesis. According to trityl assay, the stepwise yield was 99.7%. This indicates that some ODNs were lost during CBP purification and other steps, which is reasonable. It is remarkable that we were successful in using only 1/1600 of the total ODNs we synthesized and purified by CBP (theoretically 1.5625 pmol) for PCR amplification and conversion of ssODN to dsODN, and subsequent cloning and gene expression experiments. Among three colonies originated from the 399 and 401 nt ODNs that were sequenced, one contained the correct sequence for each case. This success rate, which is about 30%, is not irrationally away from the value of 55% predicted in the above analysis.

To demonstrate protein synthesis using genes constructed from the long synthetic ODNs, the pF1K vector containing the 800 bp GFP gene construct derived from the CBP purified 399 and 401 nt ODNs was transformed into *E. coli* KRX cells. Green fluorescence was observed upon induction using rhamnose (Figure 4). Without rhamnose induction, no fluorescence was observed. The gene expression results were identical to those using the control 800 bp GFP gene from a commercial source. Therefore, the complete process from automated long ODN synthesis, ODN purification by CBP, correct sequence selection by cloning and sequencing, gene construction, sequence confirmation by sequencing to gene expression is successfully established.

In theory, using long ODNs for gene and genome construction can save significant amount of reagent and solvent for ODN synthesis because using short ODNs, the gene and genome would need to be covered by ODNs almost two times due to the need of overlaps between adjacent ODNs. In contrast, using long ODNs only negligible portions require double coverage. In terms of reagent consumption and timing, the type of synthesizer makes a significant difference. In our cases, an ABI 394 synthesizer was used. For the 0.1 µmol 400 nt ODN synthesis, in which standard 1 µmol synthetic cycles with slight modifications that did not affect reagent consumption were used, less than 2 grams of each phosphoramidite were needed. The solvent acetonitrile constituted a significant portion of the cost, but about 3 liters were sufficient. Other reagent costs were minimal. The 400 nt ODN syntheses were accomplished within 60 hours. We also tried the syntheses on a MerMade 6 synthesizer. Because this synthesizer uses longer waiting time and batchwise washing as opposed to continuous flushing to save solvents, the long ODN syntheses required much longer time although solvent consumption was lower. In the future, if both reducing chemical consumption and shortening synthesis time are intended, microfluidic synthesizers may be considered.

The ability to synthesize 400 nt ODNs de novo on an automated synthesizer represents significant progress in the field of long ODN synthesis. This method has the potential to resolve long-standing problems in fields such as synthetic biology [1–2] and protein engineering [3–4]. Further increasing the length would need to address several challenges. First, the average stepwise yield needs to be above 99.8%, which is not unachievable. In our experience, when the ODNs grow longer beyond 30 nt, it is typical that the average stepwise yield increases steadily. The typical values we frequently observed were 99.6–99.8%. Assuming 99.8%, for 500 nt and 1,000 nt ODN syntheses, the total yields would be 37% and 14%, respectively. As a result, with the CBP method, which can extract minute quantities of the desired ODN product from complex mixtures, the yield is not a limiting factor for long ODN synthesis if the targets are not much longer than 1,000 nt. Second, to maintain high average stepwise yields as the ODNs grow longer, the problem of steric hindrance needs to be addressed. Our syntheses were conducted in the pores of CPG. Even though we reduced the loading to address the steric hindrance problem, there is a limit to this approach. Carrying out the syntheses on surface can be an option, but the quantity of ODN can be low [29–31]. Third, the error rate in the ODN product needs to be reduced. According to the above analysis, under standard ODN synthesis conditions, for a 400 nt ODN, the chance for an ODN molecule in the product purified by CBP to have correct sequence is 55%. For a 1,000 nt ODN synthesis, the chance would be reduced to 22%. This is still acceptable because statistically one out of four colonies containing the ODN isolated with CBP would contain the correct sequence. However, to synthesize even longer ODNs, more research is needed to reduce the error rate. If that is difficult to achieve, enzymatic correction of incorrect sequences could likely be an option although when the ODNs reach certain lengths the correction procedure itself may introduce errors to an unacceptable level [32]. More research is needed to address these problems.

## Conclusion

In summary, the de novo chemical synthesis of long ODNs with up to 400 nucleotides has been achieved. To our knowledge, these are the longest ODNs that have ever been synthesized continuously on an automated synthesizer. The ability of the catching-by-polymerization (CBP) purification method to extract minute quantities of a full-length ODN from the complex mixture generated by the thousands of reactions required for the synthesis is crucial for the success. The identity of the ODNs is confirmed by Sanger sequencing, and the pathway from long ODN synthesis and purification, correct sequence selection, gene construction to protein synthesis has been established. Because the present long ODN synthesis method does not need PCR assembly or ligation of short ODNs and secondary structures of ODNs are prevented by protecting groups during de novo synthesis, it is expected to be able to overcome many of the challenges, which include but are not limited to construction of genes with long repeats or complex secondary structures, currently faced by research projects in emerging areas such as synthetic biology and protein engineering.

## Supporting Information

File 1 Experimental details, ODN sequences, PCR primers, and sequence alignments.
