# Supplementary material for: Long oligodeoxynucleotides: chemical synthesis, isolation via catching-by-polymerization, verification via sequencing, and gene expression demonstration
Source: Beilstein J Org Chem. 2023 Dec 21;19:1957–65. doi: 10.3762/bjoc.19.146 (PMC10760481; doi:10.3762/bjoc.19.146)
Supplement: File 1 — Experimental details, ODN sequences, PCR primers, and sequence alignments. [file Beilstein_J_Org_Chem-19-1957-s001.pdf]

BEILSTEIN JOURNAL OF ORGANIC CHEMISTRY

Supporting Information

for

Long oligodeoxynucleotides: chemical synthesis, isolation via catching-by-polymerization, verification via sequencing, and gene expression demonstration

Yipeng Yin, Reed Arneson, Alexander Apostle, Adikari M. D. N. Eriyagama,
Komal Chillar, Emma Burke, Martina Jahfetson, Yinan Yuan and Shiyue Fang

Beilstein J. Org. Chem. **2023,** *19,* 1957–1965. doi:10.3762/bjoc.19.146

Experimental details, ODN sequences, PCR primers, and sequence alignments

License and Terms: This is a supporting information file under the terms of the Creative Commons Attribution License (https://creativecommons.org/licenses/by/4.0). Please note that the reuse, redistribution and reproduction in particular requires that the author(s) and source are credited and that individual graphics may be subject to special legal provisions.
The license is subject to the *Beilstein Journal of Organic Chemistry* terms and conditions: (https://www.beilstein-journals.org/bjoc/terms)

Table of contents

Experimental section S2–S5
ODN sequences S6
Primer sequences S7
Sequence alignments S8–S11
References S11

Experimental section

General information: ODNs were synthesized on an ABI 394 DNA/RNA synthesizer. PCRs were performed using an Applied Biosystems 2720 Thermal Cycler. Gel images were obtained on a UVP GelDoc-IT Imaging System 2UV Transilluminator at 302 nm. Compounds **1b** and **2** were synthesized following reported procedures [1,2]. Reagents for ODN synthesis were purchased from Glen Research and Hongene Biotech. Other small molecule chemicals such as DBU, saturated NH_4OH, *N,N*-dimethylacrylamide, *N,N'*-methylenebisacrylamide, ammonium persulfate, and *N,N,N',N'*-tetramethylethylenediamine (TMEDA) were purchased from Aldrich or other chemical companies. Authentic GFP gene and primers were purchased from IDT. GelRed® was purchased from Biotium. Solid-phase reversible immobilization (SPRI) bead (cat# CNGS005) was purchased from BullDog Bio. QIAprep® Spin Miniprep Kit, and QIAquick® Gel Extraction Kit were purchased from Qiagen. dNTPs, Phusion Hot Start II High Fidelity DNA polymerase (cat# F549S), DreamTaq^{TM} Hot Start Green PCR Master Mix (cat # K9021), and Zero Blunt™ TOPO™ PCR Cloning Kit (cat# 450031) were purchased from ThermoFisher. LB was purchased from Sigma-Aldrich. PmeI and SgfI restriction enzymes, pF1k T7 Flexi® vector (cat# C8451), and Single Step (KRX) Competent Cells (cat# L3002) were purchased from Promega. SOC medium (cat # B8020S), NEBuilder® HiFi DNA Assembly kit (cat # E5520S), and NEB® 5-alpha cells (cat # C2987H) were purchased from New England Biolab. L-Rhamnose monohydrate (cat # R-105-50) was purchased from GoldBio. Sanger sequencing was carried out at Azenta Life Sciences/Genewiz.

Chemical synthesis of long ODNs: The 800-mer GFP gene construct (all sequences are given in this supporting information) was divided into a 399 and 401 nt ODNs. They were synthesized using phosphoramidite chemistry in commercial CPG columns (0.2 µmol, 2000 Å). Before the synthesis, the columns were subject to capping conditions (see below) for 20 min. The loading of the columns was reduced to 0.1 µmol using a solution containing 0.05 M 5'-DMTr-dT amidite (**1a**) and 0.05 M 5'-Bz-dT amidite (**1b**) for the coupling step in the first synthetic cycle. Deblocking: DCA (2% in DCM), 98 s. Coupling: Bz-dA, Ac-dC, iBu-dG or dT amidite (0.1 M in ACN); DCI (0.25 M in ACN), 2.5 s × 2 reagent delivery, 35 s waiting. Capping: Cap A, THF/pyridine/acetic anhydride; cap B, 1-methylimidazole (16% in THF), 10 s delivery, 10 s waiting. Oxidation: I_2 (0.02 M in THF/pyridine/H_2O), 8 s delivery, 15 s waiting. The last nucleotide was incorporated with the polymerizable tagging amidite **2** (0.1 M in ACN, 0.25 M DCI in ACN, 5 min × 3; followed by capping and oxidation as usual; no detritylation). The CPG was divided into 10 equal portions. Deprotection and cleavage: To one portion of the CPG (theoretically 10 nmol ODN) in a 1.5 mL centrifuge tube was added DBU (10% in ACN). The tube was gently shaken at rt for 10 min. The supernatant was removed, and the DBU treatment was repeated one time. Saturated NH_4OH (0.5 mL) was added. The tube was sealed and heated at 60 °C overnight. After cooling to rt, the supernatant was transferred to a clean centrifuge tube. The CPG was washed with water (200 µL × 3). The supernatant and washes were combined, and the volume was adjusted to 200 µL by evaporation and dilution; 50 µL were used in the next steps.

Purification of long ODNs using catching-by-polymerization (CBP): Polymerization: To the 50 µL ODN solution containing **3** and **4** (Scheme 1) as well as other materials (theoretically 2.5 nmol ODN) in a 1.5 mL centrifuge tube were added 12 µL polymerization solution (*N,N*-dimethylacrylamide, 340.5 µL, 3.32 mmol; *N,N'*-methylenebisacrylamide, 17 mg, 0.11 mmol; sodium acrylate, 3 mg, 0.032 mmol; and water, 170.5 µL). After mixing, ammonium persulfate (0.23 M, 5 µL) and *N,N,N',N'*-tetramethylethylenediamine (TMEDA, 0.69 M, 5 µL) were added. The tube was closed and the content was quickly mixed with a short vortex and spin, and let stand at rt for 1 h. Washing: The gel (≈ 100 µL in size) was transferred into a 50 mL centrifuge tube. NaOAc solution (20%, pH unadjusted, 20 mL) was added.

The mixture was gently shaken at rt overnight. The gel was taken to another 50 mL centrifuge tube, and Et_3N solution (5%, 20 mL) was added. The tube was shaken at rt overnight. The gel was taken out, placed into a 2 mL centrifugal filter unit over the filter, and cut into small pieces using a spatula. The gel was washed with water (0.5 mL × 5). *Cleavage*: Minimum AcOH (80%, ≈ 200 µL) that could cover the gel was added. The mixture was incubated at rt for 5 min with occasional shaking. The liquid and gel were separated by centrifugation. The liquid was diluted with water (800 µL). The acid treatment was repeated two times. The gel was washed with water (0.3 mL × 3). The bottom of the filtering unit holding the gel was stopped with parafilm, and to the gel was added water (500 µL). The mixture was shaken at rt overnight. The solution and gel was separated by centrifugation, and the gel was washed with water (250 µL × 2). These gave ODN solutions in five tubes (≈ 1 mL each). The solutions were concentrated, combined, and evaporated to dryness. *Precipitation*: To the ODN was added NH_4OH (saturated, 50 µL). After mixing by a short vortex and spin, *n*-BuOH (450 µL) was added. The content was mixed with a vortex (≈ 20 s), and then centrifuged at 14.5k rpm (14.1k*g*) for 5 min. The supernatant was removed. The precipitated ODN (theoretically 2.5 nmol ODN) was dissolved in water (200 µL). The solution was divided into four equal portions, and all were diluted to 200 µL (theoretically 0.625 nmol). This gave the solutions of CBP-purified ODN. A portion of the solution (5 µL out of 200 µL, theoretically 15.625 pmol) was quantified to have about 40 ng ODN with a Qubit fluorometer. The cleavage and precipitation steps should be carried out within one day to avoid potential ODN damage under acidic conditions.

Conversion of long ODNs to dsODNs using PCR: The mixture of CBP-purified ODN (399 or 401 nt, 0.5 out of 200 µL, theoretically 1.5625 pmol, ≈ 20 ng as measured with UV), Phusion Hot Start II High Fidelity DNA polymerase (0.4 U), forward and reverse primers (**s1a-b** for 399 nt ODN, **s1c-d** for 401 nt ODN, 0.5 µM), HF buffer (1×), dNTPs (0.2 mM), and nuclease-free water (20 µL total mixture volume, concentrations were final) was subjected to the following touchdown PCR (TD-PCR) cycles. For the 399 nt ODN: 98 °C for 1 min for initial denaturing; 98 °C for 7 s, 70 °C (1 °C lower for each of the nine subsequent cycles) for 15 s, and 72 °C for 20 s for 10 cycles; 98 °C for 7 s, 60 °C for 15 s, and 72 °C for 20 s for 25 cycles; and 72 °C for 7 min. For the 401 nt ODN: 98 °C for 1 min for initial denaturing; 98 °C for 7 s, 68.2 °C (1 °C lower for each of the nine subsequent cycles) for 15 s, and 72 °C for 20 s for 10 cycles; 98 °C for 7 s, 58.2 °C for 15 s, and 72 °C for 20 s for 25 cycles; and 72 °C for 7 min.

Sequence analysis by comparing with authentic dsODNs: Authentic 399 and 401 bp dsODNs were obtained with PCR using commercial 800 nt GFP gene (10 ng for each reaction) as template, and **s1a-b** (for 399 nt ODN) and **s1c-d** (for 401 nt ODN) as primers, under the same conditions for converting the 399 and 401 nt ssODNs to dsODNs. The PCR products from the synthesized 399 and 401 nt ODN templates were compared with the authentic ODNs using agarose gel (1%) electrophoresis (GelRed® staining). Gel images are given in Figures 2A-B.

ODN cloning and sequencing: The PCR products of the 399 and 401 nt ODNs (45 µL out of 60 µL from three 20 µL PCR reactions) were purified with solid-phase reversible immobilization (SPRI) beads following the manufacturer's recommended procedure using a ratio of bead/sample of 1:1 (v/v). The ODNs were eluted from the beads with water (20 µL). A portion (4 µL) was used for incorporating the ODNs into pCR™4Blunt-TOPO™ vector using Zero Blunt™ TOPO™ PCR Cloning Kit (the kit allows blunt-end ligation) following the manufacturer's protocol. This gave a 6 µL vector solution. A portion (4 µL) was used to transform NEB® 5-alpha cells. The cells were plated on an agar plate containing 50 µg/mL kanamycin and grown overnight. Colony PCR with M13 primers (**s1k-l**) was performed over selected colonies that grew to amplify the insert cloned into the vector. The PCR products were analyzed on a 2% agarose gel (Figure 3). Three colonies for each of the 399 and 401 bp ODN cases that were among the ones having the shortest migration distance were selected for Sanger sequencing. For this purpose, the selected

colonies were cultured in LB containing 50 µg/mL kanamycin with shaking at 225 rpm at 37 °C overnight. Plasmid DNAs were extracted with QIAprep® Spin Miniprep Kit following the manufacturer's protocol, and submitted for Sanger sequencing. Forward and reverse reads were obtained using the M13 forward (**s1k**) and M13 reverse (**s1l**) primers, respectively. Forward and reverse reads were assembled into a single contig using the Cap3 program (https://doua.prabi.fr/software/cap3). These contigs were aligned to the reference sequence using BLAST alignment software, and aligned all together with a reference sequence using CLUSTAL Omega (see this supporting information for sequence alignments).

Construction of GFP gene: Gibson assembly was used to assemble the 399 and 401 bp dsODNs, which code the GFP expression construct, into the pF1k T7 Flexi® expression vector. *Expression vector preparation*: For cutting the pF1k T7 Flexi® vector with the PmeI and SgfI restriction enzymes, the mixture of nuclease-free water (10.5 µL), 5× Flexi buffer (4 µL), Flexi enzyme blend (0.5 µL), and pF1k T7 Flexi® vector (5 µL, 500 ng) was incubated at 37 °C for 2 h. DNA loading dye (6x, 5 µL) was added, and the mixture was heated at 65 °C for 10 min to stop the reaction. The entire content was loaded onto a 1% agarose gel for electrophoresis. The gel was stained with GelRed®. The bands corresponding to ≈ 3.1k bp were cut and purified using QIAquick® Gel Extraction Kit. The product was stored at −80 °C until use. *GFP gene ODN fragments preparation*: The plasmids containing the confirmed 399 and 401 bp ODNs were subjected to PCR using primers **s1e-f** and **s1g-h**, respectively. High Fidelity (HF) buffer (1×), dNTPs (0.2 mM, all concentrations were final), forward primer (**s1e** or **s1g**, 0.5 µM), reverse primer (**s1f** or **s1h**, 0.5 µM), plasmid (15 ng), and Phusion Hot Start II High Fidelity DNA polymerase (0.4 U) were mixed, and the volume was brought to 20 µL with nuclease-free water. For both of the 399 and 401 bp ODN cases, PCR cycles were 98 °C for 30 s for initial denaturing; 98 °C for 7 s, 66.6 °C for 15 s, and 72 °C for 20 s for 33 cycles; 72 °C for 7 min for one cycle; held at 8 °C. The product was loaded onto a 1% agarose gel for electrophoresis. The bands corresponding to ≈ 400 bp were cut, and the ODNs were isolated and purified using QIAquick® Gel Extraction Kit. *Gibson Assembly, cloning and Sanger sequencing*: The two GFP fragments and the restriction enzyme treated pF1k T7 Flexi® vector were combined using the NEBuilder® HiFi DNA Assembly Kit following the manufacturer's protocol. The solution of pF1K T7 vector (0.032 pmol, 75 ng), GFP ODN fragments (PCR products of 399 and 401 bp ODNs, ≈ 0.064 pmol, ≈ 16.9 ng), NEBuilder mix (10 µL), and nuclease-free water (total mixture volume 20 µL) was incubated at 50 °C for 20 min. A portion of this mix (2 µL) was used to transform the NEB® 5-alpha chemically competent cells following the manufacturer's protocol. The cells were plated on kanamycin plates and incubated at 37 °C overnight. Plasmid DNA of eight colonies was isolated using QIAprep® Spin Miniprep Kit. Colony PCR was performed using DreamTaq™ Hot Start Green PCR Master Mix with **s1i** and **s1j** as primers. The PCR products were analyzed with 2% agarose gel electrophoresis (lane 1, Figure 2C). All colonies were confirmed to contain the expected GFP gene. Two colonies were grown overnight in LB broth containing 50 µg/mL kanamycin. Plasmid DNAs were extracted using QIAprep® Spin Miniprep Kit, and were submitted for Sanger sequencing with a forward and reverse reactions for each (**s1i** and **s1j**). Sequence contigs were assembled and aligned as described above. Both clones were confirmed to contain the correct GFP sequence, and the GFP sequence is at the planned location in the expression vector (see this supporting information for sequence alignments).

Assembly of control GFP gene into pF1k T7 Flexi® expression vector: The authentic 800 bp GFP gene was amplified using the same PCR recipe and cycling parameters described above for the 399 and 401 bp cases using primers **s1e** and **s1h**. The PCR product was purified with gel electrophoresis, and the ODN was isolated and purified using QIAquick® Gel Extraction Kit. The gel-purified ≈ 800 bp ODN and the restriction enzyme treated pF1k T7 Flexi® vector were assembled using NEBuilder® HiFi DNA Assembly Kit with the following recipe: pF1k T7 vector (0.032 pmol), ≈ 800 bp ODN PCR product (0.064 pmol), NEBuilder mix (12 µL), and nuclease-free water (total mixture volume 24 µL). The mixture was incubated

at 50 ℃ for 20 min. A portion (2 µL) was transformed into NEB® 5-alpha chemically competent cells. Colony PCR using primers **s1i** and **s1j** was performed to select positive colonies containing the GFP gene. The PCR products were analyzed with 2% agarose gel electrophoresis (lane 2, Figure 2C). Several positive colonies were cultured in LB containing kanamycin (50 µg/mL) with shaking at 37 ℃ overnight for plasmid DNA extraction. The plasmid DNAs of two colonies were isolated with QIAprep® Spin Miniprep Kit and submitted for Sanger sequencing. Forward and reverse reads (primers **s1i** and **s1j**) were generated and assembled into contig as previously described. One sequence is correct, the other has an error (sequence alignments are provided in this supporting information file).

Expression of GFP in *E. coli*: The pF1k T7 Flexi® expression vector (20 ng) confirmed to contain the correct GFP gene originated from the synthetic 399 and 401 nt ODNs was transformed into Single Step (KRX) competent cells following the manufacturer's protocol. Briefly, KRX cells were thawed on ice for 5 min. After adding the expression vector into the cell, the tube was gently flicked, and incubated on ice for 20 min. The tube was heat shocked by heating at 42 ℃ for 20 s and cooling on ice for 5 min. SOC medium (200 µL) was added, and the tube was incubated on its side with shaking (≈ 225 rpm) at 37 ℃ for 1 h. The cells were diluted 10 times with LB. A portion (100 µL) was plated on agar plates containing kanamycin (50 µg/mL) and incubated at 37 ℃ overnight. A single colony was inoculated in LB (5 mL) containing kanamycin (50 µg/mL), and grown with 275 rpm shaking at 37 ℃ overnight. A portion (100 µL) of the culture was plated on agar plates (50 µg/µL kanamycin) with or without rhamnose (0.5%). The plates were incubated at 37 ℃ overnight. Pictures (Figures 4A-B) were taken under UV light. Pictures of cells containing the correct GFP gene originated from the authentic 800 bp ODN from commercial source were obtained using the same procedure (Figures 4C-D).

ODN sequences

Complementary sequence (i.e. antisense strand) of the entire GFP coding region plus promoter and terminator segments (800 nt ODN):

(5')TAAACCCCTCCGTTTTAGAGAGGGGTTATGCTAGTTATTTGTAGAGCTCATCCATGCCATG
TGTAATCCCAGCAGCAGTTACAAACTCAAGAAGGACCATGTGGTCACGCTTTTCGTTGGGAT
CTTTCGAAAGGGCAGATTGTGTCGACAGGTAATGGTTGTCTGGTAAAAGGACAGGGCCATC
GCCAATTGGAGTATTTTGTTGATAATGGTCTGCTAGTTGAACGGATCCATCTTCAATGTTGTG
GCGAATTTTGAAGTTAGCTTTGATTCCATTCTTTTGTTTGTCTGCCGTGATGTATACATTGTGT
GAGTTATAGTTGTACTCGAGTTTGTGTCCGAGAATGTTTCCATCTTCTTTAAAATCAATACCT
TTTAACTCGATACGATTAACAAGGGTATCACCTTCAAACTTGACTTCAGCACGCGTCTTGTA
GTTCCCGTCATCTTTGAAAGATATAGTGCGTTCCTGTACATAACCTTCGGGCATGGCACTCTT
GAAAAAGTCATGCCGTTTCATATGATCCGGATAACGGGAAAAGCATTGAACACCATAAGAG
AAAGTAGTGACAAGTGTTGGCCATGGAACAGGTAGTTTTCCAGTAGTGCAAATAAATTTAAG
GGTAAGTTTTCCGTATGTTGCATCACCTTCACCCTCTCCACTGACAGAAAATTTGTGCCCATT
AACATCACCATCTAATTCAACAAGAATTGGGACAACTCCAGTGAAAAGTTCTTCTCCTTTAC
TCATATTTTTTCCTCCTTATACTTAAGCCCTATAGTGAGTCGTATTAATTCGC(3')

T7 Terminator (complementary strand, in red in the 800 nt ODN): (5')AAACCC……TGCTAG(3')

Start codon (complementary strand, in yellow in the 800 nt ODN): (5')CAT(3')

Ribosomal binding site (RBS, complementary strand, in blue in the 800 nt ODN): (5')CTCCTT(3')

T7 Promotor (complementary strand, in green in the 800 nt ODN): (5')CCTATAGTGAGTCGTATTA(3')

399 nt ODN (shaded in yellow over the 800 nt ODN): (5')TAAACCC……CAAGGG(3')

401 nt ODN (shaded in grey over the 800 nt ODN): (5')TATCAC……ATTCGC(3')

Primer sequences

399 nt ODN forward primer (**s1a**): TAAACCCCTCCGTTTTAGAGA

399 nt ODN reverse primer (**s1b**): CCCTTGTTAATCGTATCGAGT

401 nt ODN forward primer (**s1c**): TATCACCTTCAAACTTGACTT

401 nt ODN reverse primer (**s1d**): GCGAATTAATACGACTCACT

Forward primer for preparing the 399 nt sequence for Gibson assembly (**s1e**): ccactagtaataattttctttaactttagt aaggagcgatTAAACCCCTCCGTTTTAGAGAGGG

Reverse primer for preparing the 399 nt sequence for Gibson assembly (**s1f**): AGTCAAGTTTGAAGG TGATACCCTTGTTAATCGTATCGAGTTAAAAGGTATTG

Forward primer for preparing the 401 nt sequence for Gibson assembly (**s1g**): CTCGATACGATTAAC AAGGGTATCACCTTCAAACTTGACTTCAGCACG

Reverse primer for preparing the 401 nt sequence for Gibson assembly (**s1h**): gactctagaggatccccgggtacc gagctcgaattcgtttGCGAATTAATACGACTCACTATAGGGCT

pF1K-T7-Seq-F (**s1i**): CGATCCCGCGAAATTAATACGA

pF1K-T7-Seq-R (**s1j**): AGACTGGGCGGTTTTATGGA

M13 F (**s1k**): GTAAAACGACGGCCAG

M13 R (**s1l**): CAGGAAACAGCTATGAC

399 bp ODN Alignments: (The number after "contig-399-" is the lane number in Figure 3)

```
Contig-399-15          TAAACCCCTCCGTTTTAGAGAGGGGTTATGCTAGTTATTTGTAGAGCTCATCCATGCCAT    60
contig-399-13          TAAACCCCTCCGTTTTAGAGAGGGGTTATGCTAGTTATTTGTAGAGCTCATCCATGCCAT    60
contig-399-26          TAAACCCCTCCGTTTTAGAGAGGGGTTATGCTAGTTATTTGTAGAGCTCATCCATGCCAT    60
399-bp-ODN-Reference   TAAACCCCTCCGTTTTAGAGAGGGGTTATGCTAGTTATTTGTAGAGCTCATCCATGCCAT    60
                       ************************************************************

Contig-399-15          GTGTAATCCCAGCAGCAGTTACAAACTCAAGAAGGACCATGTGGTCACGCTTTTCGTTGG    120
contig-399-13          GTGTAATCCCAGCAGCAGTTACAAACTCAAGAAGGACCATGTGGTCACGCTTTTCGTTGG    120
contig-399-26          GTGTAATCCCAGCAGCAGTTACAAACTCAAGAAGGACCATGTGGTCACGCTTTTCGTTGG    120
399-bp-ODN-Reference   GTGTAATCCCAGCAGCAGTTACAAACTCAAGAAGGACCATGTGGTCACGCTTTTCGTTGG    120
                       ************************************************************

Contig-399-15          GATCTTTCGAAAGGGCAGATTGTGTCGACG-GTAATGGTTGTCTGGTAAAAGGACAGGGC    179
contig-399-13          GATCTTTCGAAAGGGCAGATTGTGTCGACAGGTAATGGTTGTCTGGTAAAAGGACAGGGC    180
contig-399-26          GATCTTTCGAAAGGGCAGATTGTGTCGACAGGTAATGGTTGTCTGGTAAAAGGACAGGGC    180
399-bp-ODN-Reference   GATCTTTCGAAAGGGCAGATTGTGTCGACAGGTAATGGTTGTCTGGTAAAAGGACAGGGC    180
                       *****************************  *****************************

Contig-399-15          CATCGCCAATTGGAGTATTTTGTTGATAATGGTCTGCTAGTTGAACGGATCCATCTTCAA    239
contig-399-13          CATCGCCAATTGGAGTATTTTGTTGATAATGGTCTGCTAGTTGAACGGATCCATCTTCAA    240
contig-399-26          CATCGCCAATTGGAGTATTTTGTTGATAATGGTCTGCTAGTTGAACGGATCCATCTTCAA    240
399-bp-ODN-Reference   CATCGCCAATTGGAGTATTTTGTTGATAATGGTCTGCTAGTTGAACGGATCCATCTTCAA    240
                       ************************************************************

Contig-399-15          TGTTGTGGCGAATTTTGAAGTTAGCTTTGATTCCATTCTTTTGTTTGTCTGCCGTGATGT    299
contig-399-13          TGTTGTGGCGAATTTTGAAGTTAGCTTTGATTCCATTCTTTTGTTTGTCTGCCGTGATGT    300
contig-399-26          TGTTGTGGCGAATTTTGAAGTTAGCTTTGATTCCATTCTTTTGTTTGTCTGCCGTGATGT    300
399-bp-ODN-Reference   TGTTGTGGCGAATTTTGAAGTTAGCTTTGATTCCATTCTTTTGTTTGTCTGCCGTGATGT    300
                       ************************************************************

Contig-399-15          ATACATTGTGTGAGTTATAGTTGTACTCGAGTTTGTGTCCGAGAATGTTTCCATCTTCTT    359
contig-399-13          ATACATTGTGTAAGTTATAGTTGTACTCGAGTTTGTGTCCGAGAATGTTTCCATCTTCTT    360
contig-399-26          ATACATTGTGTGAGTTATAGTTGTACTCGAGTTTGTGTCCGAGAATGTTTCCATCTTCTT    360
399-bp-ODN-Reference   ATACATTGTGTGAGTTATAGTTGTACTCGAGTTTGTGTCCGAGAATGTTTCCATCTTCTT    360
                       *********** ************************************************

Contig-399-15          TAAAATCAATACCTTTTAACTCGATACGATTAACAAGGG    398
contig-399-13          TAAAATCAATACCTTTTAACTCGATACGATTAACAAGGG    399
contig-399-26          TAAAATCAATACCTTTTAACTCGATACGATTAACAAGGG    399
399-bp-ODN-Reference   TAAAATCAATACCTTTTAACTCGATACGATTAACAAGGG    399
                       ***************************************
```

contig-399-26: correct sequence

contig-399-15: one single-nucleotide (dA) deletion error

contig-399-13: one dG-to-dA substitution error

401 bp ODN Alignments: (The number after "contig-401-" is the lane number in Figure 3)

```
Contig-401-6           TATCACCTTCAAACTTGACTTCAGCACGCGTCTTGTAGTTCCCGTCATCTTTGAAAGATA    60
contig-401-10          TATCACCTTCAAACTTGACTTCAGCACGCGTCTTGTAGTTCCCGTCATCTTTGAAAGATA    60
401-bp-ODN-Reference   TATCACCTTCAAACTTGACTTCAGCACGCGTCTTGTAGTTCCCGTCATCTTTGAAAGATA    60
Contig-401-20          TATCACCTTCAAACTTGACTTCAGCACGCGTCTTGTAGTTCCCGTCATCTTTGAAAGATA    60
                       ************************************************************

Contig-401-6           TAGTACGTTCCTGTACATAACCTTCGGGCATGGCACTCTTGAAAAAGTCATGCCGTTTCA    120
contig-401-10          TAGTGCGTTCCTGTACATAACCTTCGGGCATGGCACTCTTGAAAAAGTCATGCCGTTTCA    120
401-bp-ODN-Reference   TAGTGCGTTCCTGTACATAACCTTCGGGCATGGCACTCTTGAAAAAGTCATGCCGTTTCA    120
Contig-401-20          TAGTGCGTTCCTGTACATAACCTTCGGGCATGGCACTCTTGAAAAAGTCATGCCGTTTCA    120
                       **** *******************************************************

Contig-401-6           TATGATCCGGATAACGGGAAAAGCATTGAACACCATAAGAGAAAGTAGTGACAAGTGTTG    180
contig-401-10          TATGATCCGGATAACGGGAAAAGCATTGAACACCATAAGAGAAAGTAGTGACAAGTGTTG    180
401-bp-ODN-Reference   TATGATCCGGATAACGGGAAAAGCATTGAACACCATAAGAGAAAGTAGTGACAAGTGTTG    180
Contig-401-20          TATGATCCGGATAACGGGAAAAGCATTGAACACCATAAAAGAAAGTAGTGACAAGTGTTG    180
                       ************************************** *********************

Contig-401-6           GCCATGGAACAGGTAGTTTCCCAGTAGTGCAAATAAATTTAAGGGTAAGTTTTCCGTATG    240
contig-401-10          GCCATGGAACAGGTAGTTTTCCAGTAGTGCAAATAAATTTAAGGGTAAGTTTTCCGTATG    240
401-bp-ODN-Reference   GCCATGGAACAGGTAGTTTTCCAGTAGTGCAAATAAATTTAAGGGTAAGTTTTCCGTATG    240
Contig-401-20          GCCATGGAACAGGTAGTTTTCCAGTAGTGCAAATAAATTTAAGGGTAAGTTTTCCGTATG    240
                       ******************* ****************************************

Contig-401-6           TTGCATCACCTTCACCCTCTCCACTGACAGAAAATTTGTGCCCATTAACATCA-CATCTA    299
contig-401-10          TTGCATCACCTTCACCCTCTCCACTGACAGAAAATTTGTGCCCATTAACATCACCATCTA    300
401-bp-ODN-Reference   TTGCATCACCTTCACCCTCTCCACTGACAGAAAATTTGTGCCCATTAACATCACCATCTA    300
Contig-401-20          TTGCATCACCTTCACCCTCTCCACTGACAGAAAATTTGTGCCCATTAACATCACCATCTA    300
                       ***************************************************** ******

Contig-401-6           ATTCAACAAGAATTGGGACAACTCCAGTGAAAAGTTCTTCTCCTTTACTCATATTTTTTC    359
contig-401-10          ATTCAACAAGAATTGGGACAACTCCAGTGAAAAGTTCTTCTCCTTTACTCATATTTTTTC    360
401-bp-ODN-Reference   ATTCAACAAGAATTGGGACAACTCCAGTGAAAAGTTCTTCTCCTTTACTCATATTTTTTC    360
Contig-401-20          ATTCAACAAGAATTGGGACAACTCCAGTGAAAAGTTCTTCTCCTTTACTCATATTTTTTC    360
                       ************************************************************

Contig-401-6           CTCCTTATACTTAAGCCCTATAGTGAGTCGTATTAATTCGC  400
contig-401-10          CTCCTTATACTTAAGCCCTATAGTGAGTCGTATTAATTCGC  401
401-bp-ODN-Reference   CTCCTTATACTTAAGCCCTATAGTGAGTCGTATTAATTCGC  401
Contig-401-20          CTCCTTATACTTAAGCCCTATAGTGAGTCGTATTAATTCGC  401
                       *****************************************
```

contig-401-10: correct sequence

contig-401-6: one single-nucleotide (dC) deletion, and one dT-to-dC and one dG-to-dA substitution

contig-401-20: one dG-to-dA substitution

800 bp ODN Alignments:

```
GFP-from-synthesized-ODNs-1      TAAACCCCTCCGTTTTAGAGAGGGGTTATGCTAGTTATTTGTAGAGCTCATCCATGCCAT     60
GFP-from-synthesized-ODNs-2      TAAACCCCTCCGTTTTAGAGAGGGGTTATGCTAGTTATTTGTAGAGCTCATCCATGCCAT     60
GFP-from-commercial-ODN-1        TAAACCCCTCCGTTTTAGAGAGGGGTTATGCTAGTTATTTGTAGAGCTCATCCATGCCAT     60
800-bp-ODN-Reference             TAAACCCCTCCGTTTTAGAGAGGGGTTATGCTAGTTATTTGTAGAGCTCATCCATGCCAT     60
GFP-from-commercial-ODN-2        TAAACCCCTCCGTTTTAGAGAGGGGTTATGCTAGTTATTTGTAGAGCTCATCCATGCCAT     60
                                 ************************************************************

GFP-from-synthesized-ODNs-1      GTGTAATCCCAGCAGCAGTTACAAACTCAAGAAGGACCATGTGGTCACGCTTTTCGTTGG     120
GFP-from-synthesized-ODNs-2      GTGTAATCCCAGCAGCAGTTACAAACTCAAGAAGGACCATGTGGTCACGCTTTTCGTTGG     120
GFP-from-commercial-ODN-1        GTGTAATCCCAGCAGCAGTTACAAACTCAAGAAGGACCATGTGGTCACGCTTTTCGTTGG     120
800-bp-ODN-Reference             GTGTAATCCCAGCAGCAGTTACAAACTCAAGAAGGACCATGTGGTCACGCTTTTCGTTGG     120
GFP-from-commercial-ODN-2        GTGTAATCCCAGCAGCAGTTACAAACTCAAGAAGGACCATGTGGTCACGCTTTTCGTTGG     120
                                 ************************************************************

GFP-from-synthesized-ODNs-1      GATCTTTCGAAAGGGCAGATTGTGTCGACAGGTAATGGTTGTCTGGTAAAAGGACAGGGC     180
GFP-from-synthesized-ODNs-2      GATCTTTCGAAAGGGCAGATTGTGTCGACAGGTAATGGTTGTCTGGTAAAAGGACAGGGC     180
GFP-from-commercial-ODN-1        GATCTTTCGAAAGGGCAGATTGTGTCGACAGGTAATGGTTGTCTGGTAAAAGGACAGGGC     180
800-bp-ODN-Reference             GATCTTTCGAAAGGGCAGATTGTGTCGACAGGTAATGGTTGTCTGGTAAAAGGACAGGGC     180
GFP-from-commercial-ODN-2        GATCTTTCGAAAGGGCAGATTGTGTCGACAGGTAATGGTTGTCTGGTAAAAGGACAGGGC     180
                                 ************************************************************

GFP-from-synthesized-ODNs-1      CATCGCCAATTGGAGTATTTTGTTGATAATGGTCTGCTAGTTGAACGGATCCATCTTCAA     240
GFP-from-synthesized-ODNs-2      CATCGCCAATTGGAGTATTTTGTTGATAATGGTCTGCTAGTTGAACGGATCCATCTTCAA     240
GFP-from-commercial-ODN-1        CATCGCCAATTGGAGTATTTTGTTGATAATGGTCTGCTAGTTGAACGGATCCATCTTCAA     240
800-bp-ODN-Reference             CATCGCCAATTGGAGTATTTTGTTGATAATGGTCTGCTAGTTGAACGGATCCATCTTCAA     240
GFP-from-commercial-ODN-2        CATCGCCAATTGGAGTATTTTGTTGATAATGGTCTGCTAGTTGAACGGATCCATCTTCAA     240
                                 ************************************************************

GFP-from-synthesized-ODNs-1      TGTTGTGGCGAATTTTGAAGTTAGCTTTGATTCCATTCTTTTGTTTGTCTGCCGTGATGT     300
GFP-from-synthesized-ODNs-2      TGTTGTGGCGAATTTTGAAGTTAGCTTTGATTCCATTCTTTTGTTTGTCTGCCGTGATGT     300
GFP-from-commercial-ODN-1        TGTTGTGGCGAATTTTGAAGTTAGCTTTGATTCCATTCTTTTGTTTGTCTGCCGTGATGT     300
800-bp-ODN-Reference             TGTTGTGGCGAATTTTGAAGTTAGCTTTGATTCCATTCTTTTGTTTGTCTGCCGTGATGT     300
GFP-from-commercial-ODN-2        TGTTGTGGCGAATTTTGAAGTTAGCTTTGATTCCATTCTTTTGTTTGTCTGCCGTGATGT     300
                                 ************************************************************

GFP-from-synthesized-ODNs-1      ATACATTGTGTGAGTTATAGTTGTACTCGAGTTTGTGTCCGAGAATGTTTCCATCTTCTT     360
GFP-from-synthesized-ODNs-2      ATACATTGTGTGAGTTATAGTTGTACTCGAGTTTGTGTCCGAGAATGTTTCCATCTTCTT     360
GFP-from-commercial-ODN-1        ATACATTGTGTGAGTTATAGTTGTACTCGAGTTTGTGTCCGAGAATGTTTCCATCTTCTT     360
800-bp-ODN-Reference             ATACATTGTGTGAGTTATAGTTGTACTCGAGTTTGTGTCCGAGAATGTTTCCATCTTCTT     360
GFP-from-commercial-ODN-2        ATACATTGTGTGAGTTATAGTTGTACTCGAGTTTGTGTCCGAGAATGTTTCCATCTTCTT     360
                                 ************************************************************

GFP-from-synthesized-ODNs-1      TAAAATCAATACCTTTTAACTCGATACGATTAACAAGGGTATCACCTTCAAACTTGACTT     420
GFP-from-synthesized-ODNs-2      TAAAATCAATACCTTTTAACTCGATACGATTAACAAGGGTATCACCTTCAAACTTGACTT     420
GFP-from-commercial-ODN-1        TAAAATCAATACCTTTTAACTCGATACGATTAACAAGGGTATCACCTTCAAACTTGACTT     420
800-bp-ODN-Reference             TAAAATCAATACCTTTTAACTCGATACGATTAACAAGGGTATCACCTTCAAACTTGACTT     420
GFP-from-commercial-ODN-2        TAAAATCAATACCTTTTAACTCGATACGATTAACAAGGGTATCACCTTCAAACTTGACTT     420
                                 ************************************************************

GFP-from-synthesized-ODNs-1      CAGCACGCGTCTTGTAGTTCCCGTCATCTTTGAAAGATATAGTGCGTTCCTGTACATAAC     480
GFP-from-synthesized-ODNs-2      CAGCACGCGTCTTGTAGTTCCCGTCATCTTTGAAAGATATAGTGCGTTCCTGTACATAAC     480
GFP-from-commercial-ODN-1        CAGCACGCGTCTTGTAGTTCCCGTCATCTTTGAAAGATATAGTGCGTTCCTGTACATAAC     480
800-bp-ODN-Reference             CAGCACGCGTCTTGTAGTTCCCGTCATCTTTGAAAGATATAGTGCGTTCCTGTACATAAC     480
GFP-from-commercial-ODN-2        CAGCACGCGTCTTGTAGTTCCCGTCATCTTTGAAAGATATAGTGCGTTCCTGTACATAAC     480
                                 ************************************************************

GFP-from-synthesized-ODNs-1      CTTCGGGCATGGCACTCTTGAAAAAGTCATGCCGTTTCATATGATCCGGATAACGGGAAA     540
GFP-from-synthesized-ODNs-2      CTTCGGGCATGGCACTCTTGAAAAAGTCATGCCGTTTCATATGATCCGGATAACGGGAAA     540
GFP-from-commercial-ODN-1        CTTCGGGCATGGCACTCTTGAAAAAGTCATGCCGTTTCATATGATCCGGATAACGGGAAA     540
800-bp-ODN-Reference             CTTCGGGCATGGCACTCTTGAAAAAGTCATGCCGTTTCATATGATCCGGATAACGGGAAA     540
GFP-from-commercial-ODN-2        CTTCGGGCATGGCACTCTTGAAAAAGTCATGCCGTTTCATATGATCCGGATAACGGGAAA     540
                                 ************************************************************

GFP-from-synthesized-ODNs-1      AGCATTGAACACCATAAGAGAAAGTAGTGACAAGTGTTGGCCATGGAACAGGTAGTTTTC     600
GFP-from-synthesized-ODNs-2      AGCATTGAACACCATAAGAGAAAGTAGTGACAAGTGTTGGCCATGGAACAGGTAGTTTTC     600
GFP-from-commercial-ODN-1        AGCATTGAACACCATAAGAGAAAGTAGTGACAAGTGTTGGCCATGGAACAGGTAGTTTTC     600
800-bp-ODN-Reference             AGCATTGAACACCATAAGAGAAAGTAGTGACAAGTGTTGGCCATGGAACAGGTAGTTTTC     600
GFP-from-commercial-ODN-2        AGCATTGAACACCATAAGAGAAAGTAGTGACAAGTGTTGACCATGGAACAGGTAGTTTTC     600
                                 *************************************** ********************

GFP-from-synthesized-ODNs-1      CAGTAGTGCAAATAAATTTAAGGGTAAGTTTTCCGTATGTTGCATCACCTTCACCCTCTC     660
GFP-from-synthesized-ODNs-2      CAGTAGTGCAAATAAATTTAAGGGTAAGTTTTCCGTATGTTGCATCACCTTCACCCTCTC     660
GFP-from-commercial-ODN-1        CAGTAGTGCAAATAAATTTAAGGGTAAGTTTTCCGTATGTTGCATCACCTTCACCCTCTC     660
800-bp-ODN-Reference             CAGTAGTGCAAATAAATTTAAGGGTAAGTTTTCCGTATGTTGCATCACCTTCACCCTCTC     660
GFP-from-commercial-ODN-2        CAGTAGTGCAAATAAATTTAAGGGTAAGTTTTCCGTATGTTGCATCACCTTCACCCTCTC     660
                                 ************************************************************
```

```
GFP-from-synthesized-ODNs-1     CACTGACAGAAAATTTGTGCCCATTAACATCACCATCTAATTCAACAAGAATTGGGACAA     720
GFP-from-synthesized-ODNs-2     CACTGACAGAAAATTTGTGCCCATTAACATCACCATCTAATTCAACAAGAATTGGGACAA     720
GFP-from-commercial-ODN-1       CACTGACAGAAAATTTGTGCCCATTAACATCACCATCTAATTCAACAAGAATTGGGACAA     720
800-bp-ODN-Reference            CACTGACAGAAAATTTGTGCCCATTAACATCACCATCTAATTCAACAAGAATTGGGACAA     720
GFP-from-commercial-ODN-2       CACTGACAGAAAATTTGTGCCCATTAACATCACCATCTAATTCAACAAGAATTGGGACAA     720
                                ************************************************************

GFP-from-synthesized-ODNs-1     CTCCAGTGAAAAGTTCTTCTCCTTTACTCATATTTTTTCCTCCTTATACTTAAGCCCTAT     780
GFP-from-synthesized-ODNs-2     CTCCAGTGAAAAGTTCTTCTCCTTTACTCATATTTTTTCCTCCTTATACTTAAGCCCTAT     780
GFP-from-commercial-ODN-1       CTCCAGTGAAAAGTTCTTCTCCTTTACTCATATTTTTTCCTCCTTATACTTAAGCCCTAT     780
800-bp-ODN-Reference            CTCCAGTGAAAAGTTCTTCTCCTTTACTCATATTTTTTCCTCCTTATACTTAAGCCCTAT     780
GFP-from-commercial-ODN-2       CTCCAGTGAAAAGTTCTTCTCCTTTACTCATATTTTTTCCTCCTTATACTTAAGCCCTAT     780
                                ************************************************************

GFP-from-synthesized-ODNs-1     AGTGAGTCGTATTAATTCGC     800
GFP-from-synthesized-ODNs-2     AGTGAGTCGTATTAATTCGC     800
GFP-from-commercial-ODN-1       AGTGAGTCGTATTAATTCGC     800
800-bp-ODN-Reference            AGTGAGTCGTATTAATTCGC     800
GFP-from-commercial-ODN-2       AGTGAGTCGTATTAATTCGC     800
                                ********************
```

The two GFP genes from synthesized ODNs are correct. For the two control GFP genes from commercial ODN, one is correct, and the other has a dG-to-dA substitution error.

References

1. Azhayev, A.; Gouzaev, A.; Hovinen, J.; Azhayeva, E.; Lonnberg, H. Analogs of oligonucleotides containing 3'-deoxy-β-D-Psicothymidine. *Tetrahedron Lett.*, **1993**, *34*, 6435-6438.
2. Pokharel, D.; Fang, S. Y. Polymerizable phosphoramidites with an acid-cleavable linker for eco-friendly synthetic oligodeoxynucleotide purification. *Green Chem.*, **2016**, *18*, 1125-1136.
